# Improvement in Renal Hemodynamics following Combined Angiotensin II Infusion and AT1R Blockade in Aged Female Sheep following Fetal Unilateral Nephrectomy

**DOI:** 10.1371/journal.pone.0068036

**Published:** 2013-07-01

**Authors:** Reetu R. Singh, Yugeesh R. Lankadeva, Kate M. Denton, Karen M. Moritz

**Affiliations:** 1 School of Biomedical Sciences, The University of Queensland, St Lucia, Queensland, Australia; 2 Department of Physiology, Monash University, Clayton, Victoria, Australia; 3 Department of Anatomy and Developmental Biology, Monash University, Clayton, Victoria, Australia; The University of Manchester, United Kingdom

## Abstract

Renin-angiotensin system (RAS) is a powerful modulator of renal hemodynamic and fluid homeostasis. Up-regulation in components of intra-renal RAS occurs with ageing. Recently we reported that 2 year old uninephrectomised (uni-x) female sheep have low renin hypertension and reduced renal function. By 5 years of age, these uni-x sheep had augmented decrease in renal blood flow (RBF) compared to sham. We hypothesised that this decrease in RBF in 5 year old uni-x sheep was due to an up-regulation in components of the intra-renal RAS. In this study, renal responses to angiotensin II (AngII) infusion and AngII type 1 receptor (AT1R) blockade were examined in the same 5 year old sheep. We also administered AngII in the presence of losartan to increase AngII bioavailability to the AT2R in order to understand AT2R contribution to renal function in this model. Uni-x animals had significantly lower renal cortical content of renin, AngII (∼40%) and Ang 1–7 (∼60%) and reduced cortical expression of AT1R gene than sham animals. In response to both AngII infusion and AT1R blockade via losartan, renal hemodynamic responses and tubular sodium excretion were significantly attenuated in uni-x animals compared to sham. However, AngII infusion in the presence of losartan caused ∼33% increase in RBF in uni-x sheep compared to ∼14% in sham (P<0.05). This was associated with a significant decrease in renal vascular resistance in the uni-x animals (22% vs 15%, P<0.05) without any changes in systemic blood pressure. The present study shows that majority of the intra-renal RAS components are suppressed in this model of low renin hypertension. However, increasing the availability of AngII to AT2R by AT1R blockade improved renal blood flow in uni-x sheep. This suggests that manipulation of the AT2R maybe a potential therapeutic target for treatment of renal dysfunction associated with a congenital nephron deficit.

## Introduction

Hypertension is prevalent in 25% of the adult population and contributes to the development of coronary heart disease, stroke and chronic kidney disease [1]. An abnormal kidney development resulting in a reduced nephron number has been suggested to be a risk factor for chronic renal insufficiency and hypertension [2]. However, long-term follow up studies in kidney donors, (in whom there is rapid removal of half the number of nephrons in adult life), have shown that only a minority of donors develop proteinuria, a decline in glomerular filtration rate (GFR) or elevation in arterial pressure [3], [4]. Conversely, children who are born with a solitary kidney have been shown to have an increased incidence of hypertension and microalbuminuria [5] and a very recent study showed that the median age for developing renal injury in children with congenital absence of a kidney was as young as 15 years [6]. Many experimental studies have shown an association between a nephron deficit from birth to development of hypertension in adult offspring that experienced perturbations such as maternal dietary protein restriction [7], [8], [9] or elevations in the levels of maternal stress hormones; glucocorticoids, *in-utero* [10], [11], [12]. Collectively, the clinical and experimental findings suggest that a reduction in nephron number from birth or very early in life is a significant risk factor for future renal and cardiovascular diseases. A caveat, however, to most of the aforementioned experimental studies is that many prenatal perturbations also cause fetal growth restriction [7], [13], [14], which can independently predispose to cardiovascular disease in developmental programming models [15], [16]. This makes it difficult to appreciate the contribution of a nephron deficit per se in the development of hypertension in these models.

In order to overcome this hurdle and to understand the mechanisms through which a nephron deficit induced *in-utero* contributes to renal dysfunction and hypertension, our group has established a model of fetal uninephrectomy (uni-x) in sheep [17], [18]. Development of the permanent (metanephric) kidney in sheep [19] is very similar to that in human [20] with both species completing nephrogenesis prior to birth. We have previously reported that fetal uni-x in this model results in a 30% reduction in nephron number rather than 50% due to compensatory nephrogenesis in the remaining kidney [17]. Our previous studies in male sheep showed that uni-x sheep had low renin hypertension, lower sodium excretion, albuminuria [21] and 30% reduction in glomerular filtration rate (GFR) and renal blood flow (RBF) from 6 months of age compared to sham animals [22]. The renal dysfunction and hypertension exacerbated with ageing in these male sheep [23].

In many models of low renin hypertension, an up-regulation of the intra-renal renin-angiotensin system (RAS) has been reported to contribute to impaired renal function and maintenance of the systemic hypertension (for a review see, [24]. Certainly, many developmental programming models of hypertension have shown an up-regulation in components of the intra-renal RAS [14], [25], [26], [27]. However, studies in our model showed that 6 month old low-renin hypertensive, uni-x male sheep had significantly lower levels of renal renin and AngII content together with reduced renal gene expression of AT1R and AT2R compared to sham [22]. Recently, in response to an acute period of volume expansion via 0.9% saline loading (25 ml/kg/40 mins) we observed a blunted decrease in plasma renin activity (PRA) in 5 year old low-renin hypertensive female uni-x sheep [28]. The reasons for the attenuated decrease in PRA in response to volume expansion in the uni-x females maybe simply due to the already low basal PRA [28], however based on the aforementioned studies an up-regulation of the intra-renal RAS as observed in other low renin models of hypertension may also be an underlying mechanism. Therefore, we hypothesised that these aged uni-x female sheep with low-renin hypertension may have an increase in expression and function of components of intra-renal RAS. While this hypothesis is contrary to our observation in male sheep [22], sex differences in renal and cardiovascular responses to infusion of Angiotensin II (AngII), AngII type 1 receptor (AT1R) blockade and AT2R stimulation have previously been reported with the differences associated at least in part to greater AT2R expression and function in females [29], [30], [31]. Furthermore, Schulman et al [32] have reported a decline in PRA levels but an up-regulation in components of the intra-renal RAS with ageing.

Therefore to investigate whether the intra-renal RAS was upregulated in aged female sheep, we examined renal and cardiovascular responses to systemic AngII and losartan (AT1R blockade) infusion in 5 year old sham and uni-x female sheep. We also examined the renal expression of major components of the RAS. Since sex differences in renal function have been demonstrated to be associated with AT2R expression [33], in the present study we also examined the contribution of the AT2R to renal and cardiovascular function by performing AngII infusion in the presence of losartan (AT1R blockade) in 5 year old female sheep.

## Materials and Methods

### Ethics Statement

Experiments were performed in pure-bred Australian merino sheep following approval from an Animal Ethics Committee of Monash University. All experiments were conducted according to guidelines of the National Health and Medical Research Council of Australia.

### Cohort Set-up

Two-three year old merino ewes were mated and the first day of conception denoted as gestational age 0. At 100 days post-conception, surgery was performed where ewes and fetuses were anaesthetised with sodium pentothal (1 g I.V.) and maintained on halothane (1.5–2% in O_2_). In five singleton female fetuses, the left renal artery, vein and ureter were ligated and the left kidney was excised (fetal uni-x group). In five other singleton female fetuses, the left kidney was cleared from surrounding fat but was not excised (sham control group). At 5 months of age lambs underwent surgery, where the right carotid artery was surgically exteriorised into skin fold to form a carotid arterial loop [34]. At 5 years of age, animals were brought into the laboratory, placed in individual metabolic cages and allowed a week to acclimatise to laboratory conditions. All animals were maintained on a diet of hay and chaff for the duration of their stay in the laboratory.

### Measurement of Cardiovascular and Renal Function

Following the acclimatisation period, animals were instrumented with a carotid arterial catheter (Tygon cannula) for measurement of mean arterial pressure (MAP) and heart rate (HR) and two venous catheters (right and left jugular vein) for infusion purposes. For determination of renal function a Foley catheter, (Size 12, French, Bardia Malaysia) was inserted into the bladder of all animals for continuous collection of urine. Glomerular filtration rate (GFR) was determined via the clearance of ^51^Cr-ethylenediaminetetraacetic acid (10 ml bolus of 15 µCi, followed by intravenous infusion at 15 µCi/h) and effective renal plasma flow (ERPF), and hence renal blood flow (RBF), were determined via clearance of para-aminohippurate (PAH) (PAH, 4.8 mg/kg/l in 10 ml bolus, followed by intravenous infusion at 750 mg/h); these were infused at a combined rate of 12 ml/h as previously described [35]. PAH concentration was determined using rapid microplate assay method [36]. Renal vascular resistance (RVR) was determined as [MAP/RBF]. Filtration fraction was determined as [(GFR/ERPF) × 100]; urinary sodium excretion (U_Na_V) was determined as [urine flow rate (UFR) × urinary sodium concentration]; filtered load of sodium (FL_Na_) was calculated as [plasma sodium concentration × GFR] and percentage fractional sodium excretion (FE_Na_ %) was calculated as [(U_Na_V/filtered load of sodium) × 100].

### Response to Exogenous AngII, AT1R Blockade and AngII in the Presence of AT1R Blockade

On the day of experimentation, allowing an hour for ^Cr^51EDTA and PAH to equilibrate, cardiovascular and renal function was determined over a 1 hour period to establish baseline (basal period 1; B1). Following this the response to RAS was determined in three stages: Stage 1; AngII phase; infusion of a low pressor dose of AngII (0.2 µg/kg/h i.v) for a 90 minute period followed by a 1 hour recovery period to establish a new baseline (basal period 2; B2) prior to commencement of Stage 2: losartan phase; losartan was administered as a 10 ml bolus (0.95 mg/kg) and then an infusion of losartan was maintained at (1.9 mg/kg/h i.v.), Stage 3: AngII in the presence of losartan phase; after 90 minutes of losartan infusion, animals were re-administered the low pressor dose of AngII for 90 minutes during which losartan infusion was maintained. Plasma ^51^Cr EDTA and PAH were allowed to reach steady-state for the first 30 minutes of each infusion period, thus data analysis was performed using measurements obtained in the last hour of each drug infusion stage. For comparisons for data analysis, response to AngII was compared to B1, response to losartan was compared to B2 and response to AngII in the presence of losartan was compared to losartan as the baseline.

### Determination of Renal and Systemic Components of RAS

Three weeks following the completion of all experiments, animals were humanely euthanized (pentobarbitone, Lethabarb®). A 0.5 cm slice was taken from one half of the right kidney, in transverse plane, and subdivided into cortex and medulla (inner and outer combined), homogenised and RNA extracted for determining gene expression of AT1R and AT2R by real-time PCR using a comparative cycle of CT (threshold fluorescence) method using 18S as the housekeeping gene as previously described [10]. Plasma and tissue levels of renin, AngII and tissue Ang 1–7 levels were also determined via radioimmunoassay (Prosearch International, Malvern, Australia).

### Statistical Analysis

Values are presented as the mean ± SEM, with level of significance set at P≤0.05. To compare differences between sham and uni-x animals, a student’s t-test was performed where stated. To examine the effect of drug infusion in each treatment group repeated measures analysis of variance followed by a multiple comparison Bonferroni post-hoc analysis was performed. A two-way analysis of variance was performed to examine the differences in gene expression or renal RAS content between the treatment groups and different regions of the kidney. Statistical analysis was performed using GraphPad Prism 5.0 (GraphPad Software, San Diego, CA).

## Results

### Birth Weight, Growth and Kidney Weights

All animals were born at term (149±1 day). Birth and body weights were not different between the treatment groups and total kidney weights and kidney weights corrected for body weights were also similar between the groups (Birth weight (kg): sham; 4.0±0.5, uni-x; 4.0±0.3, Body weight (kg) at 5 years: sham; 56±2, uni-x; 54±6, Total kidney weight: sham: (left and right) kidney weight, (g); 121±6, uni-x: right kidney weight (g); 108±6). Total kidney to body weight ratio (g/kg): sham; 2.2±0.1, uni-x; 2.1±0.2). These data for birth weight and growth and kidney weights for these animals have been previously reported [28], [37].

### Basal Hematocrit, Plasma Sodium, Renin and AngII

Plasma sodium and hematocrit levels were similar between the two groups. However, PRA and plasma AngII levels were significantly lower in the uni-x compared to the sham group (P = 0.009 and P = 0.01, for PRA and AngII, respectively, Table 1).

**Table 1:** Basal plasma electrolyte and hormones and basal cardiovascular and renal parameters in 5 year old sham and uninephrectomised (uni-x) sheep.

	Sham *(N = 5)*	Uni-x *(N = 5)*
**Plasma**		
Sodium (mmol/l)	137±0.6	136±1.1
Hematocrit (%)	25.4±0.7	24.5±0.6
Renin activity (ng/ml/h)	1.4±0.6	0.6±0.2**
AngII (pg/ml)	22.6±7.2	12.3±1.2*
**Cardiovascular**		
MAP (mm Hg)	82±1	93±1****
Heart rate (beats/min)	82±4	85±5
**Renal**		
GFR (ml/min/gkw)	1.02±0.1	0.62±0.1***
RBF (ml/min/gkw)	10.1±0.8	5.3±0.5***
RVR (mm Hg/ml/min/gkw)	8.4±0.7	17.9±1.4****
Fitration fraction (%)	13.9±1.8	16.6±2.8
UFR (ml/min/gkw)	0.01±0.005	0.01±0.002
U_Na_V (umol/min/gkw)	2.0±0.5	1.2±0.2
FE_Na_ %	1.4±0.4	1.2±0.4

* P<0.05,

** P<0.01,

*** P<0.001,

**** P<0.0001.

### Basal Cardiovascular and Renal Measurements

Basal cardiovascular and renal variables obtained over 1 hour are shown in Table 1. Heart rates were similar between the treatment groups. Uni-x animals had significantly higher basal MAP and RVR compared to the sham animals (P<0.0001 for both). Basal GFR and RBF were significantly lower in the uni-x animals (P<0.001 and P<0.001 for GFR and RBF, respectively), while filtration fraction was similar between the treatment groups. UFR was similar between the treatment groups but uni-x animals had significantly lower U_Na_V (P<0.001) compared to the sham group. FE_Na_ % was lower in the uni-x animals but this did not reach statistical significance (P = 0.09).

### Cardiovascular and Renal Response to AngII (Basal Period B1 as the Control Comparison)

**Figure 1:**
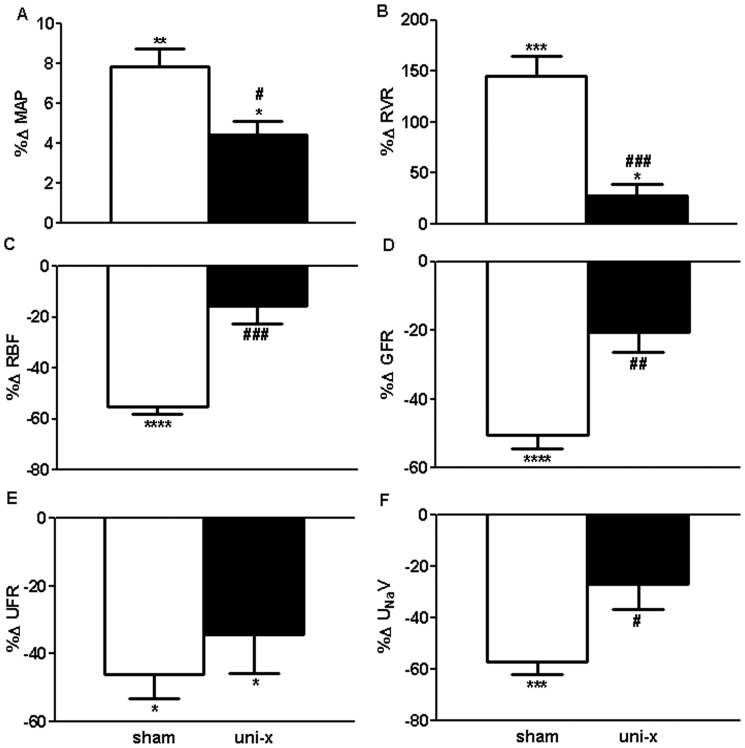
Cardiovascular and renal responses to intravenous infusion of low pressor dose angiotensin II (AngII) expressed as percentage change (%Δ) from basal period 1 in sham and uni-x female sheep at 5 years of age (n = 5/group). MAP, mean arterial pressure; RVR, renal vascular resistance; RBF, renal blood flow; GFR, glomerular filtration rate; UFR, urine flow rate; U_Na_V, urinary sodium excretion; all values except MAP are corrected for total kidney weight (gkw). Sham: white bars, uni-x: black bars, *P<0.05, ***P<0.001, ****P<0.0001 from Bonferroni post-hoc test comparing responses of sham or uni-x animals to AngII infusion from their basal period 1. ^#^P<0.05, ^##^P<0.01, ^###^P<0.001 from student’s t-test comparing sham and uni-x. Values are mean ± SEM.

MAP increased from B1 in response to AngII in both treatment groups but this increase was significantly less in the uni-x group compared to the sham animals (Increase in MAP (mmHg); sham; 6.5±0.7, uni-x; 4.1±0.6, P_interaction_ = 0.04, Figure 1A). Similarly, AngII caused an increase in RVR in both treatment groups, however this response was significantly lower in the uni-x animals compared to sham (P_interaction_ = 0.02, Figure 1B). AngII caused a significant decrease in both RBF and GFR from B1 in sham animals; it had no significant effect on RBF and GFR in the uni-x animals (RBF; P_interaction_ = 0.0004, GFR: P_interaction_ = 0.006, Figures 1C and 1D, respectively). AngII infusion had no effect on filtration fraction in either treatment group. UFR declined in response to AngII in both treatment groups similarly (P_AngII_ = 0.005, P_interaction_ = 0.4, Figure 1E). UFR declined in response to AngII in both treatment groups similarly (P_AngII_ = 0.005, P_interaction_ = 0.4, Figure 1E). However U_Na_V was significantly different between the treatment groups during AngII infusion and this was due to a decrease in U_Na_V in the sham group but not in the uni-x group (P_interaction_ = 0.007, Figures 1F). FE_Na_% was not affected by AngII infusion in either treatment group.

### Cardiovascular and Renal Responses to AT1 Blockade (Basal Period 2 (B2) as the Control Comparison)

**Figure 2:**
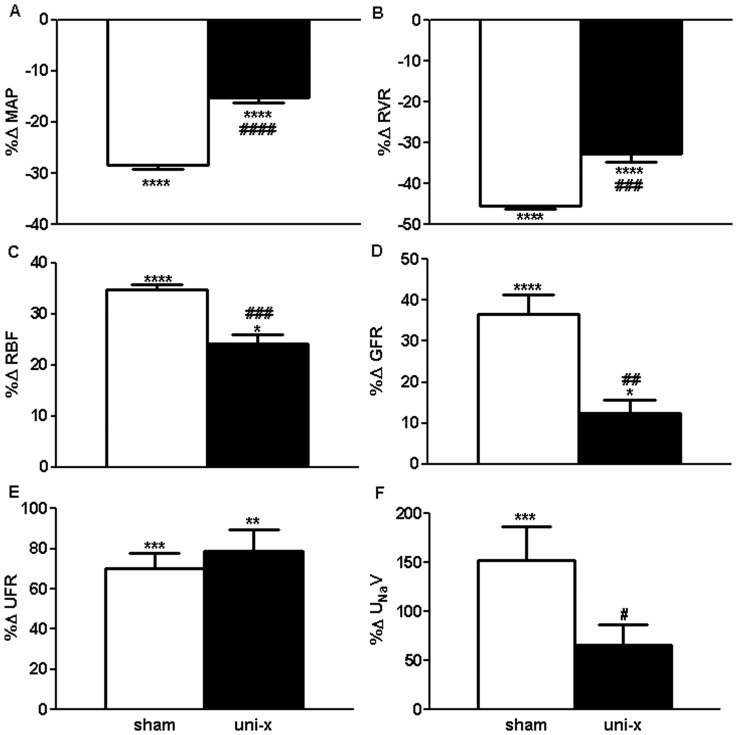
Cardiovascular and renal responses to AT1R blockade via intravenous infusion of losartan expressed as percentage change (%Δ) from basal period 2 in sham and uni-x female sheep at 5 years of age (n = 5/group). MAP, mean arterial pressure; RVR, renal vascular resistance; RBF, renal blood flow; GFR, glomerular filtration rate; UFR, urine flow rate; U_Na_V, urinary sodium excretion; all values except MAP are corrected for total kidney weight (gkw).B2, basal period 2; sham: white bars, uni-x: black bars, *P<0.05, **P<0.01, ***P<0.001, ****P<0.0001 from Bonferroni post-hoc test comparing responses of sham or uni-x animals to losartan from their basal period 2. ^#^P<0.05, ^##^P<0.01, ^###^P<0.001, ^####^P<0.0001 from student’s t-test comparing sham and uni-x.Values are mean ± SEM.

All variables returned to a similar basal level following cessation of the AngII infusion; B2 was not significantly different to B1 for any variable in either group. In response to losartan, MAP and RVR decreased in both treatment groups, however this response was attenuated in the uni-x animals (MAP; P_interaction_ = 0.0008, RVR; P_interaction_ = 0.02, Figures 2A and 2B, respectively). RBF and GFR increased in response to losartan and this increase was also significantly less in the uni-x animals compared to the sham group (RBF; P_interaction_ = 0.006, GFR; P_interaction_ = 0.0001, Figures 2C and 2D, respectively). Filtration fraction was unchanged in both treatment groups in response to losartan. UFR increased in response to losartan similarly between the treatment groups (P_Losartan_ = 0.0001, P_interaction_ = 0.2, Figure 2E). Losartan infusion caused a significant increase in U_Na_V in the sham group whereas the response was significantly diminished in uni-x animals (151% v 79%; P_interaction_ = 0.007, Figure 2F). Similarly FE_Na_% increased in response to losartan and this response tended to be greater in the sham animals compared to the uni-x (96% v 46%; P_interaction_ = 0.09).).

#### Response to AngII in the Presence of Losartan (Losartan Period as the Control Comparison)

**Figure 3:**
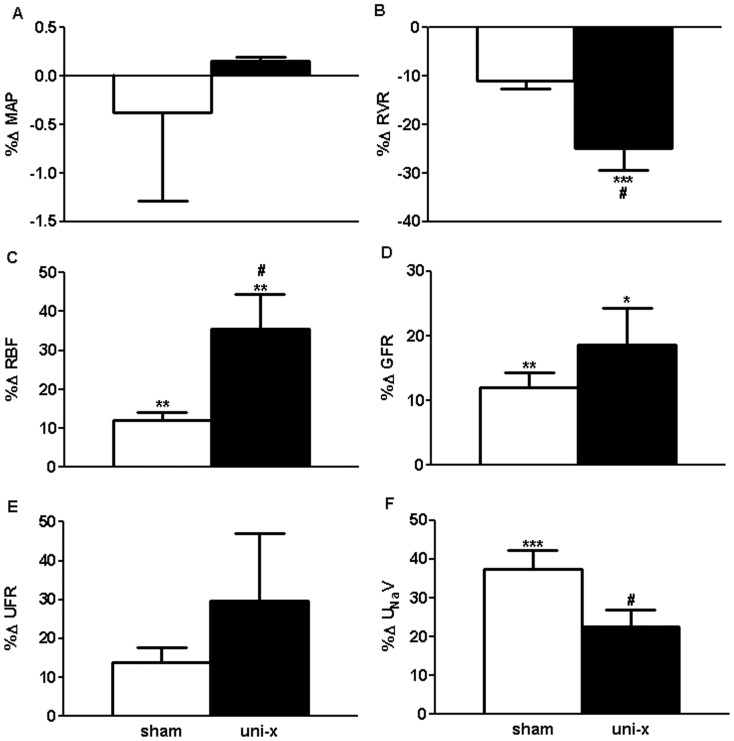
Cardiovascular and renal responses to Angiotensin II infusion in the presence of AT1R blockade via losartan expressed as percentage change (%Δ) from losartan infusion period in sham and uni-x female sheep at 5 years of age (n = 5/group). MAP, mean arterial pressure; RVR, renal vascular resistance; RBF, renal blood flow; GFR, glomerular filtration rate; UFR, urine flow rate; U_Na_V, urinary sodium excretion; all values except MAP are corrected for total kidney weight (gkw). Sham: white bars, uni-x: black bars, *P<0.05, **P<0.01, ***P<0.001, from Bonferroni post-hoc test comparing responses of sham or uni-x animals to AngII plus losartan from their losartan infusion period. Values are mean ± SEM.

AngII infusion in the presence of losartan had no effect on MAP in either treatment group (Figure 3A). However, RVR decreased to a greater extent in the uni-x animals compared to the sham (P_interaction_ = 0.01, Figure 3B). In response to AngII+Losartan, RBF and GFR increased in both treatment groups (RBF; P_AngII+Losartan_ = 0.0002, GFR; P_AngII+Losartan_ = 0.0001, Figures 3C and 3D, respectively). Post-hoc analysis revealed that whilst the percent change in GFR was similar between the sham and uni-x treatment groups (11±2% v 18±5%, respectively) the percent increase in RBF was significantly greater in the uni-x as compared to the sham animals (33±9% v 14±2%, P<0.05, Figure 3C). There was no difference in filtration fraction or UFR (Figure 3E) between the treatment groups in response to AngII+Losartan. U_Na_V and FE_Na_% increased in response to AngII infusion in the presence of losartan, however this increase in U_Na_V response was significantly less in the uni-x animals (U_Na_V; P_interaction_ = 0.008, Figure 3F, FE_Na_%; P_interaction_ = 0.1).

### Angiotensin II Receptor mRNA Expression

**Figure 4:**
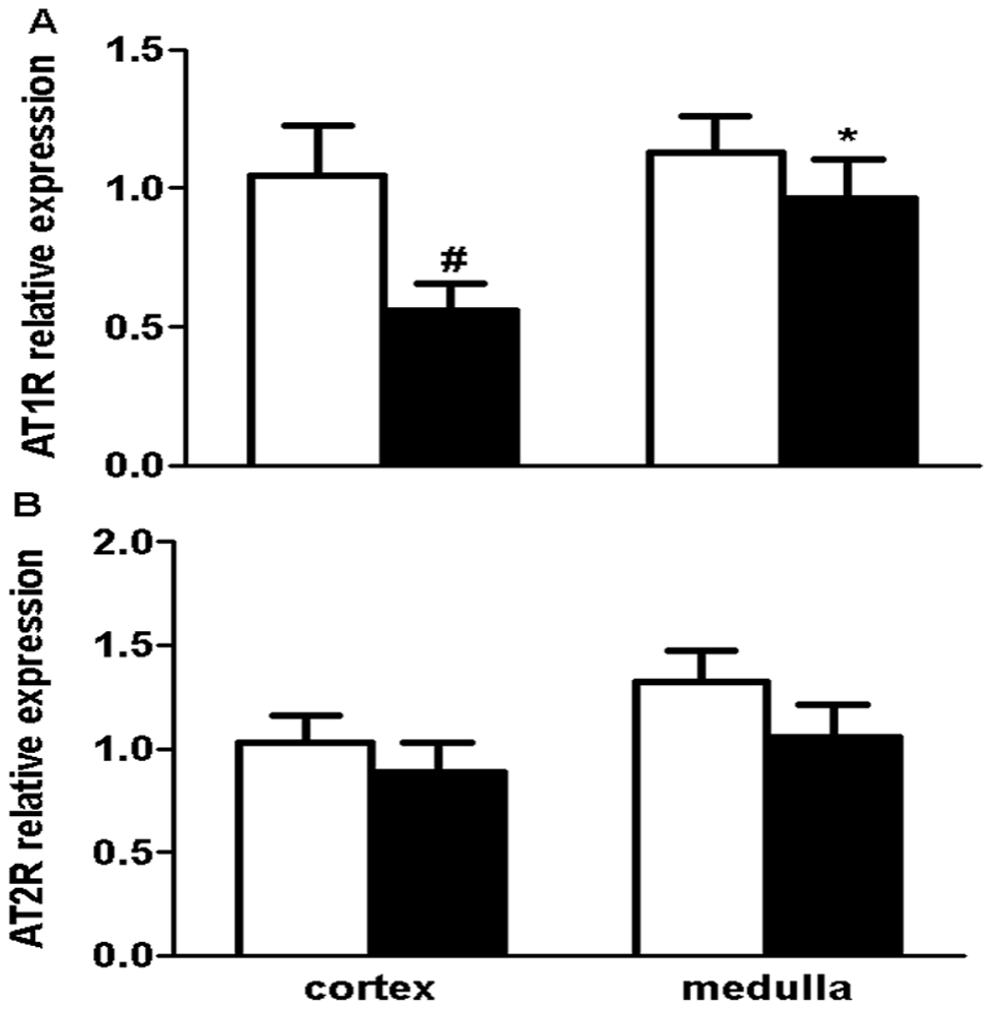
Angiotensin II receptor gene expression in the kidney cortex and medulla of 5 year old female sheep (A) Angiotensin II receptor type 1 (AT1R), (B) Angiotensin II receptor type 2 (AT2R). Sham (white bars, N = 5) and uni-x (black bars, N = 5) Values are mean ± SEM. ^#^P<0.05 comparing sham and uni-x animals and *P<0.05 comparing kidney zones within the sham or uni-x group from Bonferroni post-hoc test.

AT1R gene expression was significantly lower in the kidney cortex of the uni-x group compared to the sham (P_group_ = 0.048, Figure 4A) while AT1R gene expression in the medulla was not different between the treatment groups. Cortical and medullary AT1R gene expression was not different between the sham animals, however uni-x animals had higher AT1R gene expression in the medulla compared to their cortex (P<0.05). AT2R mRNA expression was similar between the treatment groups in both the kidney cortex and medulla (Figure 4B). The ratio of AT2R:AT1R was greater in the uni-x kidney cortex compared to sham, whilst expression in the medulla was not different between the groups (AT2R/AT1R; cortex; sham: 1.01±0.09, uni-x: 1.64±0.14, P = 0.006 from t-test, medulla; sham: 0.86±0.06, uni-x: 0.92±0.07).

### Renal Renin, AngII and Ang 1–7 Content

**Figure 5:**
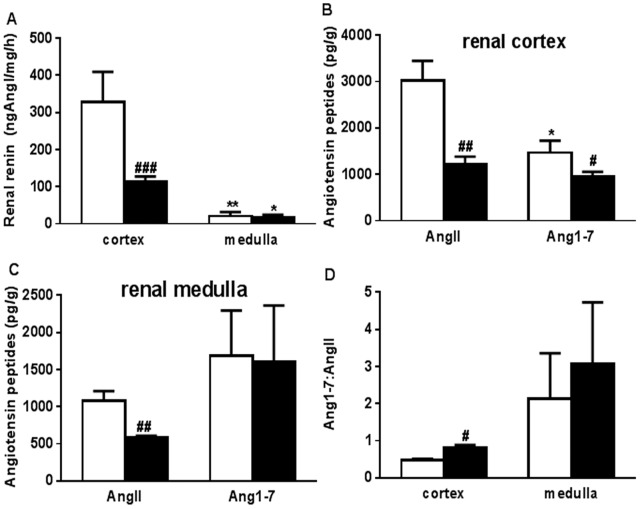
Renal tissue (A) renin content, angiotensin peptide (Angiotensin II (AngII), Angiotensin 1–7 (Ang 1–7) content in the renal (B) cortex and (C) medulla, and (C) Angiotensin 1–7:AngII ratio in sham and uni-x kidneys at 5 years of age. Sham (white bars, N = 5) and uni-x (black bars, N = 5). Values are mean ± SEM. ^#^P<0.05, ^##^P<0.01, ^###^P<0.001 comparing sham and uni-x groups and *P<0.05, **P<0.01, comparing kidney zones within the sham and uni-x group from Bonferroni post-hoc test.

Renal renin content was significantly lower in the kidney cortex of uni-x animals than sham group but did not differ between the groups in the medulla (P_group_ = 0.047, P_kid.zone_ = 0.0005, P_group_ ×_ kid.zone_ = 0.02, Figure 5A). Overall renin levels in the medulla were less than cortex in both groups. Renal AngII levels were significantly lower in both the kidney cortex (P = 0.004, Figure 5B) and the medulla (P = 0.008, Figure 5C) of the uni-x animals compared to the sham group. Ang 1–7 content was also significantly lower in uni-x kidney cortex compared to sham kidney cortex (P = 0.03, Figure 5B). In the sham animals cortical Ang1–7 was lower than their cortical AngII content (P = 0.01, Figure 5B) whilst in the uni-x animals, cortical AngII and Ang 1–7 levels were not different. While Ang1–7 levels in the medulla were similar between the treatment groups they varied significantly within each group and were not different to medullary AngII levels for either treatment group (Figure 5C). The ratio of Ang1–7 to AngII was significantly greater in the uni-x kidney cortex compared to the sham kidney cortex (Ang 1–7/AngII; cortex; sham: 0.04±0.003, uni-x: 0.08±0.009, P = 0.02, Figure 5D). While Ang1–7/AngII ratio was not different between the treatment groups in the medulla, there was significant variation in levels within the groups.

## Discussion

The main findings of the present study were that in response to AngII infusion and AT1R blockade, renal hemodynamic and the arterial pressor responses were significantly attenuated in 5 year old uni-x female sheep. However, when AngII was infused in the presence of AT1R blockade, thereby increasing AngII availability to bind to the AT2R, uni-x sheep had a greater decrease in RVR and a greater increase in RBF than sham animals. This improvement in RBF associated with a decrease in RVR may be due in part to the greater Ang1–7/AngII or AT2R/AT1R mRNA ratios in the uni-x animals. Furthermore, our data suggests that while the predominant responses due to AngII binding to the AT1R may overshadow the effects of AT2R, stimulation of the AT2R in the presence of AT1R blockade may be beneficial in improving renal function in this model of reduced nephron endowment.

Arterial pressor responses to AngII were modest (∼6 mmHg in sham and ∼4 mmHg in uni-x) and this increase in arterial pressure was significantly attenuated in the female uni-x sheep compared to sham animals. This finding is consistent with our observation in uni-x male sheep [22]. The attenuated increase in MAP in response to AngII in the uni-x animals could be due to the already reduced levels of circulating renin and AngII. Another possibility is that the diminished MAP response is associated with a reduced increase in cardiac output in the uni-x animals. Recently, we have reported that cardiac functional reserve is significantly impaired in these same uni-x female sheep in response to a cardiac challenge [37]. In contrast to the modest differences in pressor responses between the treatment groups, renal hemodynamic responses to AngII were markedly different between the treatment groups. Whilst RVR increased and GFR and RBF decreased in both treatment groups, these responses were significantly attenuated in the uni-x animals. These responses are likely associated with the differences in cortical expression of components of the intra-renal RAS, specifically the reduced content of renin, AngII and reduced gene expression of AT1R in the female uni-x sheep. The reduced AT1R mRNA expression in the renal cortex of uni-x sheep has likely resulted in reduced AngII binding and thus lessened the vasoconstrictive effects (increase in RVR and decrease in RBF) of AngII in the uni-x sheep. We acknowledge that gene and protein expression do not always correlate but we have not measured protein levels due to lack of specific commercial antibodies [38] but we are confident reporting mRNA levels for the present study, particularly because they support our functional findings. Uni-x sheep also exhibited a blunted natriuretic response to AngII compared to the sham sheep and this response is likely associated with the attenuated GFR response in uni-x sheep resulting in lower filtered load delivered for excretion. The finding of a down-regulated intra-renal RAS is consistent with our observations in young male uni-x sheep [22] and in rat offspring with a reduced nephron endowment induced by maternal protein-restriction [14].

In response to AT1R blockade, MAP and RVR decreased, whilst RBF and GFR increased in both treatment groups. However, the extents of these responses were significantly attenuated in the uni-x animals. Furthermore, while UFR increased similarly in both groups, AT1R blockade blunted the increase in U_Na_V in the uni-x animals. The current observations in these older female uni-x sheep are in contrast to our observation in young male uni-x sheep in which a greater decrease in MAP and a greater increase in U_Na_V occurred following losartan infusion [22]. This increase in U_Na_V during AT1R blockade in the uni-x male sheep was postulated to reflect an enhanced intratubular RAS [22]. The differences likely indicate differential response to AT1R blockade in the male and female uni-x sheep as has been observed in humans. For example, 4 weeks of AngII receptor blockade (ARBS) has similar effects on blood pressure and renal hemodynamics in healthy men and women [39], whilst in hypertensive subjects ARBS produced a greater decrease in diastolic pressure in young women as compared to men [40]. Experimental studies have also shown an enhanced depressor response to losartan in young female spontaneously hypertensive rat (SHR) than aged-match male rats whilst in the aged SHR female rats, this depressor response is blunted [41]. It is plausible that the responses to AT1R blockade may have been enhanced in young female uni-x sheep similar to or even to a greater extent to what was observed in young uni-x male sheep [22], however with increasing age, this responsiveness has diminished. Certainly in the same cohort of animals we have previously reported that plasma renin levels declined significantly between 1 to 5 years of age in the uni-x female sheep compared to sham [37]. Thus, it is possible that the attenuated decrease in MAP response following AT1R blockade in these aged female sheep is associated with the reduced systemic RAS thus reduced stimulation of the AT1R. Future studies need to investigate the responses to AT1R blockade in young uni-x female sheep to establish if the responses indeed change with the ageing process. In the present study, levels of AngII were significantly lower in the uni-x animals both in the systemic circulation and within the kidney cortex and medulla; thus the diminishing in systemic and renal hemodynamic and tubular responses to AT1R blockade are in accord with the reduction in both the systemic and renal RAS in the uni-x female sheep.

While the reduction in plasma renin levels in the uni-x animals is appropriate given the elevation in arterial pressure, it is unclear why the intra-renal RAS is low in the hypertensive uni-x animals. Particularly since clinically the elevation in arterial pressure in low-renin hypertensives has been shown to be maintained by an inappropriately activated intra-renal RAS [42]. The decrease in endogenous renal renin content maybe directly associated with the reduction in nephron number and as a result, reduced numbers of juxtaglomerular cells in the uni-x sheep kidney. Furthermore, differing mechanisms of renal ablation appear to differentially influence the expression of the intra-renal RAS, where in the surgical excision model of reduced renal mass (uninephrectomy), it has been shown that renal renin content is significantly reduced compared to the infarction model (uninephrectomy plus artery ligation of the remaining kidney), in which renal renin content is significantly increased [43]. Another finding of the present study that differs from some models of hypertension is that pressor responses to AngII infusion were significantly attenuated in the uni-x animals compared to the sham sheep. While these findings are consistent with observation in uni-x male sheep [22], they are dissimilar to some reports in programming models of reduced nephron number and hypertension which have reported augmented responses to AngII [44], [45], [46]. Interestingly, responses to AngII appear to be different in even similar developmental programming models depending on tissue types examined. For example, in the case of maternal glucocorticoid treatment, a reduction in nephron number in the offspring has been observed following treatment with betamethsone [47], dexamethasone [48] and cortisol [49]. However, while enhanced pressor response to intravenous AngII infusion have been reported in male offspring prenatally exposed to betamethasone [44] pressor responses to AngII in prenatally dexamethasone and cortisol exposed male offspring have been reported to be unaltered [50]. In contrast, central AngII infusion (intracerebroventricular) caused increased pressor responsiveness in animals exposed prenatally to dexamethasone but not in those exposed to cortisol [51]. Therefore it is likely that differences in expression and responses of the RAS in models of reduced nephron endowment are due to the multiplicity of models employed, and the sex and age at which offspring are studied and highlight that renal programming of nephron number and/or hypertension may not have a unifying pathway in all models.

AngII infusion in the presence of losartan was performed to investigate the contribution of the AT2R to renal function and blood pressure regulation in this model. AngII infusion in the presence of losartan had no effect on arterial pressure in the present study similar to studies of direct AT2R stimulation in the rat [29] and to observations in hypertensive models [52]. Previously, AT2R stimulation has been reported to decrease RVR and filtration fraction and increase RBF, urine flow and urinary sodium excretion in female rats; effects that were abolished in the presence of AT2R blockade [29]. In the present study, AngII infusion in the presence of losartan caused an increase in GFR, RBF, U_Na_V and a decrease in RVR which is consistent with AT2R stimulation study in the female rat [29], validating that our protocol caused sufficient activation of the AT2R to alter renal hemodynamics. Surprisingly the increase in RBF and the decrease in RVR were significantly greater whilst the increase in U_Na_V was significantly less in the uni-x animals, in the presence of AngII plus losartan infusion than what was observed for losartan infusion alone. These renal responses in the presence of AngII+Losartan indicate that blockade of the AT1R together with infusion of AngII was necessary to cause sufficient stimulation of the AT2R to exert its effects on renal hemodynamics. In addition to AngII, AT2R can be stimulated via Ang1–7 and cause vasodilation and natriuresis [53]. For the first time we report while absolute renal content of Ang1–7 was significantly lower in the renal cortex of uni-x animals consistent with observations in untreated essential hypertensive subjects [54], the proportion of basal expression of Ang1–7 to AngII was significantly greater in the uni-x animals compared to the sham kidney cortex whilst levels in medulla were not different. This higher basal ratio in the uni-x animals may be due to either a greater degradation of AngII or greater formation rate of Ang1–7. In the present study the greater increase in RBF and decrease in RVR in the uni-x female sheep in response to AngII infusion in the presence of AT1R blockade suggests a greater stimulation of the AT2R in the uni-x animals and this may be associated with the greater expression of AT2R:AT1R mRNA ratio in the uni-x animals. The slightly higher Ang1–7/AngII content ratio in the uni-x kidney cortex may also provide an alternate route for AT2R stimulation. Studies with specific receptor agonist are required in the future to examine this finding in detail.

### Limitations

The present study has revealed a decrease in RVR and increase in RBF in response to AngII infusion in the presence of AT1R blockade; this suggests activation of the AT2R in the present study. A limitation of the present study is that we did not investigate direct AT2R stimulation as has been performed in rats [29] which given the size of the sheep and the cost of these agonists was not feasible for the present study. Another limitation of the present study is that we cannot at the moment delineate whether the beneficial effects observed are due to AT2R stimulation or Mas-receptor stimulation. While blockade of the AT1R increases the availability of AngII to AT2R, AngII can also be converted to Ang 1–7 and exert hemodynamic effects via the Mas-receptor [53]. While our findings suggest that “direct” AT2R stimulation via specific agonists may significantly improve renal function in subjects with renal dysfunction associated with a congenital nephron deficit, future studies are needed to affirm this. Another limitation of the present study is that we did not have age-matched male sheep to perform similar studies in therefore cannot affirm that the beneficial effects observed in females in response to AngII plus losartan infusion apply to male sheep as well. The increases in ratio of AT2R:AT1R and Ang1–7/AngII are in themselves intriguing and raise the question of whether these changes are a compensatory response to protect against further progression of renal dysfunction or a direct result of the fetal uni-x. Future studies need to be undertaken to examine the profile of the RAS in young to ageing animals.

### Conclusion

A congenital nephron deficit induced by fetal unilateral nephrectomy in female sheep results in significant suppression of the renal and systemic RAS at 5 years of age. Despite this, an increase in ratios of intra-renal Ang 1–7/AngII and AT2R/AT1R was observed in the uni-x animals and may indicate an elevation in the second arm of the RAS in female animals with a reduced nephron endowment. Whilst responses to AngII infusion and AT1R blockade were significantly attenuated in the uni-x animals most likely due to reduced AT1R expression, increased AT2R stimulation via combined AngII infusion and AT1R blockade elicited beneficial effects on reducing renal vascular resistance and improving renal blood flow in the uni-x animals.
